# Response to Forker et al

**DOI:** 10.1038/s44318-024-00141-1

**Published:** 2024-06-21

**Authors:** Simonne Griffith-Jones, Lucía Álvarez, Urbi Mukhopadhyay, Sarah Gharbi, Mandy Rettel, Michael Adams, Janosch Hennig, Sagar Bhogaraju

**Affiliations:** 1 European Molecular Biology Laboratory, 71 avenue des Martyrs, 38042 Grenoble, France; 2 European Molecular Biology Laboratory, Meyerhofstraße 1, 69117 Heidelberg, Germany; 3 Biochemistry IV, Biophysical Chemistry, University of Bayreuth, Universitätsstrasse 30, 95447 Bayreuth, Germany

**Keywords:** Post-translational Modifications & Proteolysis, Structural Biology

## Abstract

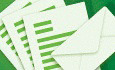

Forker et al (this issue) reported the crystal structure of the MAGE-A4 MAGE-homology domain in complex with the RAD6-binding domain (R6B) of the ubiquitin ligase RAD18. The reported crystal structure and our AlphaFold (AF) predicted model (Griffith-Jones et al, 2024) differ with respect to the orientation of the RAD18 R6B helix interacting with MAGE-A4. However, we agree with Forker et al that both the crystal structure and the AlphaFold model concur on two crucial aspects: the interaction sites on both proteins and the specific residues involved in the interaction. In this context, it is important to highlight that we used AlphaFold primarily to generate a structural hypothesis and later went on to use that to gain insights into what this interaction means for both proteins in cells and in vitro. In addition, our NMR, pulldown, and ITC data can independently implicate the exact region and residues in MAGE-A4 involved in RAD18 interaction. And most importantly, all the NMR, biochemical, and functional data reported in our study (Griffith-Jones et al, 2024) provide context to both the crystal structure and the AlphaFold model.

Below, we discuss our findings in the context of the new crystal structure reported by Forker et al. In addition, we explore this unique scenario where experimental methods typically used for AlphaFold structural validation might not provide a complete picture, especially when the AlphaFold model agrees with the experimental structure on some crucial aspects, as in the case of MAGE-A4/ RAD18 R6B complex.

(1) In our manuscript, we reported the AlphaFold-predicted model of MAGE-A4 MHD bound to RAD18 R6B. Since our attempts to crystallize this complex were unsuccessful, we turned to AlphaFold to test if it provides a model that we can validate using other experimental approaches. We used both the classical version of AlphaFold (Jumper et al, 2021) with MAGE-A4 MHD fused to the RAD18 R6B, and AlphaFold multimer (preprint: Evans et al, 2022) where individual polypeptide sequences were provided as input. Since the highest-ranking models in both scenarios reported similar structures, we reported only one. As Forker et al, point out, their attempts to perform AlphaFold also resulted in similar models as ours. Our NMR experiments (Fig. 1H,I in (Griffith-Jones et al, 2024)) show chemical shift perturbations in MAGE-A4 that are compatible with both the AlphaFold model and the experimental structure reported by Forker et al. So, the NMR data supported the AF model and fell short of revealing the orientation flip of RAD18 R6B that is reported in the latest experimental structure. As Forker et al write in their correspondence, “*Despite the flipped helix orientation, there is considerable overlap of the interfacial residues of the MAGEA4 MHD between the crystal structure and the AlphaFold models (compare Fig. 1B with Fig. 1D in this manuscript and Fig. 1D in* (Griffith-Jones et al, 2024)*; shared surface residues from MAGEA4 MHD are labeled in blue in Fig. 1BD*)”. We agree with this observation. It is worth noting that even after the R6B helix flipping, the spot of interaction in MAGE-A4 and the face of the RAD18 R6B helix contacting MAGE-A4 in the predicted AF model are in alignment with the crystal structure. Therefore, the MAGEA4 residues involved in binding could be correctly inferred in our study (Griffith-Jones et al, 2024). Interestingly, in our data involving pulldown experiments and ITC (Fig. 1E–G in (Griffith-Jones et al, 2024)), we showed a loss of binding to RAD18 when specific residues in MAGE-A4 were mutated. These mutated residues were designed based on the AlphaFold model, and they resulted in loss of binding to RAD18 because these residues are indeed involved in the complex interface even according to the crystal structure reported by Forker et al. It is of utmost importance that we highlight here that our NMR experiments, interpreted alone, can lead to similar structural conclusions as the crystal structure reported by Forker et al (see below for details).

Overall, this discrepancy is yet another reminder that AlphaFold-predicted models, although having the potential to provide key insights into biology, may be inaccurate to varying degrees and will be corrected by experimental structures down the line (Terwilliger et al, 2024). As of now, apart from pLDDT scores and PAE scores, which are widely accepted and which we also report in our manuscript (Fig. EV1A,B in (Griffith-Jones et al, 2024)), there is no other silver bullet to assess the accuracy of these models. To make matters a little more complicated in our specific case, our experimental approaches—NMR, ITC, and pulldowns—missed this incorrect orientation of RAD18 R6B peptide because the AlphaFold-predicted model was accurate enough that it showed us the correct interacting regions of MAGE-A4/RAD18 and even pointed us to specific residues involved in binding. This is an interesting case study for the structural biology community in general to highlight how AlphaFold models, while providing highly useful hypotheses, might still differ from experimental structures. We think this issue of AlphaFold is especially relevant for small hydrophobic protein-protein interfaces, where the relative orientation of molecules might not make a big difference to model-quality indicator scores such as PAE. On the other hand, in our specific case, we still have to be open to the possibility that the short helix of RAD18 R6B might be involved in dynamic interactions with MAGE-A4 by adopting varying orientations, two of which are captured by AlphaFold and crystallography.

(2) As a prelude to the second point below, we would like to point to an AlphaFold commentary by experts in structural biology (https://www.embl.org/news/science/alphafold-potential-impacts/), which says “*As with experimental structures, predicted structures may (or may not) lead to hypotheses about the function of the protein and the mechanism underlying that function, but such hypotheses then have to be tested by further experimentation*.”

Here, we would like to provide functional context to both the crystal structure reported by Forker et al, and the structural data we have described in our study (Griffith-Jones et al, 2024) based on AlphaFold models and NMR data:

## Figure 1: The RAD18 R6BD binds to the C-terminal WH-B motif of MAGEA4

The pulldown experiments (Fig. 1E), ITC (Fig. 1F,G), and the NMR experiments (Fig. H,I) were all carried out independently of the AlphaFold model and can be used to infer that RAD18 interacts with the WH-B subdomain of MAGE-A4. In addition, our NMR chemical shift perturbation (CSP) data shown in Fig. EV1 (panel I) (and the pulldown experiments in Fig. 1E together) implicate MAGE-A4 residues M161, I205, V239, V291, and V294 in RAD18-R6B interaction. These exact residues are also pointed out by Forker et al in their communication of the crystal structure. So, in conclusion, our experimental data clearly reveal the exact region in MAGE-A4 to which RAD18 binds, and also correctly highlight specific residues involved through NMR CSPs, pulldown, and ITC experiments.

## Figure 2: The “dileucine motif” of MAGEA4 contributes towards MAGEA4 stability

Here, we used crystal structures, point mutations, and size-exclusion chromatography to demonstrate the importance of the dileucine motif for the stability of MAGEA4.

## Figure 3: MAGEA4 causes conformational changes in the RAD18/RAD6 complex

Here, we used a range of techniques, including pulldowns, isothermal calorimetry, density gradient centrifugation, cross-linking mass spectrometry, and mass photometry to show that the RAD18/RAD6 complex undergoes conformational changes upon MAGEA4 binding, and elucidated the stoichiometry of binding between these proteins. In Fig. 3A, we used the AlphaFold model to show the imminent clashes with RAD6 and MAGE-A4 if they were both competing for binding to RAD18 (Fig. 4). These clashes could also be independently predicted from our NMR data (Figs. 1 and EV1). In addition, these clashes would still occur according to the crystal structure reported by Forker et al. In Fig. 5C, we did use mutants of MAGEA4, which were designed based on the AlphaFold model. However, the residues mutated (M161 and I205) were also implicated in MAGE-A4/RAD18 interaction based on our pulldown experiments, ITC and NMR (Fig. 1E–I and EV1). In addition, these residues are also shown to be involved in the MAGE-A4/RAD18 interaction in the crystal structure reported by Forker et al.

## Figure 4: Interactions between RING and SAP domains of RAD18 are essential for PCNA monoubiquitination

These data do not concern the MAGE-A4 and RAD18 interaction but rather inform about specific intramolecular interactions within RAD18. Here, we used a different AlphaFold model to predict the interaction between the RING and SAP domains of RAD18. We validated this intramolecular interface in RAD18 thoroughly by ubiquitination assays in vitro and in cells using UV-induced RAD18 puncta formation.

## Figure 5: MAGEA4 inhibits RAD18 autoubiquitination without hindering PCNA ubiquitination

Here, we used in vitro ubiquitination assays to demonstrate that MAGE-A4 inhibits RAD18 autoubiquitination activity. In Fig. 5B,C, we did use mutants of MAGEA4, which were designed based on the AlphaFold model. Importantly, the residues mutated (M161 and I205) were also implicated in the MAGE-A4/RAD18 interaction based on our pulldown experiments, ITC, and NMR (Figs. 1E–I and EV1). Furthermore, the residues mutated (M161 and I205) were shown to be involved in the MAGE-A4/RAD18 interaction in the crystal structure reported by Forker et al. The claims made through this figure are supported by orthogonal experiments involving cross-linking mass spectrometry and di-Gly site identification in RAD18 using mass spectrometry (Fig. 5D,E).

## Figure 6: Identification of a potential “ligase-binding cleft” in type-I MAGEs

Here our hypothesis of a potential ligand-binding cleft in MAGEs originated from the AlphaFold model of MAGE-A4 and RAD18 R6B independently corroborated by the NMR, pulldown, and ITC experiments (Figs. 1 and EV1). It is important to note that the crystal structure of MAGE-A4 and RAD18 R6B reported by Forker et al also implicates this cleft in MAGEA4 for binding the RAD18. So, our hypothesis that there might be a common ligase-binding cleft in MAGEs remains accurate. In this figure, we then went on to test this hypothesis in MAGE-C2, where we made a single point mutation facing the ligase-binding cleft and showed the loss of binding to TRIM28 in cells using a quantitative mass spectrometry experiment (Fig. 6D,E).

In summary, none of the conclusions of our study (Griffith-Jones et al, 2024) are affected by the discrepancies between the crystal structure and the AlphaFold model. However, it needs to be highlighted, as Forker et al do in their communication, that structural biologists have to be aware of this odd feature of AlphaFold, by which it predicts the regions and residues involved in the interaction relatively accurately but reorients the helix as in this case. As mentioned above, we think this characteristic of AlphaFold is probably more relevant for small hydrophobic protein-protein interfaces, where the relative orientation of molecules might not make a big difference to the AlphaFold model-quality indicator scores such as PAE. It is undeniable that these are still relatively early days for AlphaFold integration into structural biology, and this will surely evolve as cases like this emerge. As this important paper (Terwilliger et al, 2024) puts it perfectly, “AlphaFold predictions are valuable hypotheses and accelerate but do not replace experimental structure determination”.
